# Hyperbranched Silicone MDTQ Tack Promoters

**DOI:** 10.3390/molecules24224133

**Published:** 2019-11-15

**Authors:** Sijia Zheng, Shuai Liang, Yang Chen, Michael A. Brook

**Affiliations:** Department of Chemistry and Chemical Biology, McMaster University, 1280 Main St. W., Hamilton, ON L8S 4M1, Canada; zhengs16@mcmaster.ca (S.Z.); shuailiang618@gmail.com (S.L.); dychen@mcmaster.ca (Y.C.)

**Keywords:** silicone dendrimer, precise MDTQ resins, tacky silicone gels, plasticization vs. swelling, branched v linear fluids

## Abstract

Low molecular weight, highly crosslinked silicone resins are widely used as reinforcing agents for highly transparent elastomers and adhesion/tack promoters in gels. The resins are complex mixtures and their structure / property relationships are ill defined. We report the synthesis of a library of 2, 3 and 4-fold hyperbranched polymeric oils that are comprised of linear, lightly branched or highly branched dendronic structures. Rheological examination of the fluids and tack measurements of gels filled with 10, 25 or 50% dendronic oils were made. Viscosity of the hyperbranched oils themselves was related to molecular weight, but more significantly to branch density. The properties are driven by chain entanglement. When cured into a silicone gel, less densely branched materials were more effective in improving tack than either linear oils or Me_3_SiO-rich, very highly branched oils of comparable molecular weight, because the latter oils underwent phase separation.

## 1. Introduction

Silicone resins are highly reticulated materials comprised primarily of tri- and/or tetrafunctional SiO monomers and can be considered to be 3-dimensional hyperbranched copolymers. In industry, MQ resins are of particular interest (the common nomenclature used for silicones is Q: SiO_4/2_; T: MeSiO_3/2_; D: MeSiO_2/2_ and M: Me_3_SiO~) [1]. MQ resins are highly reticulated silica-like cores capped with hydrophobic Me_3_Si groups. Practical resins are usually relatively low molecular weight polymers (<10,000 g mol^−1^) that are typically surface capped to remove ‘sticky’ SiOH groups using M (MeSiO) groups, or functional Me groups, including SiMe_2_H and Si Me_2_Vinyl (Si-CH=CH_2_). As a consequence of their small silica particle-like structure, they are widely used as reinforcing fillers to impart an improvement of the mechanical properties of highly transparent silicone elastomers [2], and may also be used as viscosity modifiers, adhesion/tack additives, and passivating layers in electronics [2,3].

**Figure 1:**
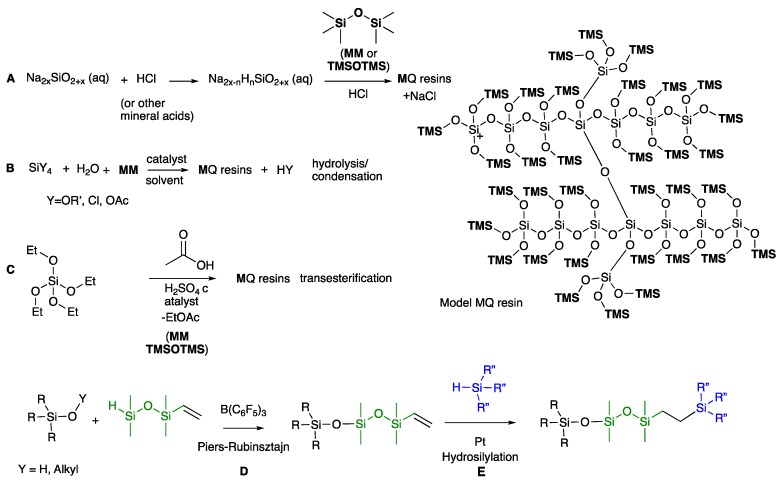
Simple preps of MQ resins by (**A**) acidification of sodium silicate following by capping; (**B**) co-hydrolysis; (**C**) transesterification. (**D**) example of the Piers-Rubinsztajn reaction; (**E**) example of Pt-catalyzed hydrosilylation.

MQ resins are an important class of commercial silicone resins. They can be prepared in a sol-gel process by acidification of sodium silicate followed by capping [4], or hydrolysis and condensation [5]; recently, a transesterification approach to their synthesis using Si(OEt_4_) was described (Figure 1A–C) [6].

MQ resins, because of their ability to act as silicone reinforcing agents, have been widely employed in the applications of personal care product such cosmetic products (e.g., eye shadow, sun cream, antiperspirant stick, face lotion, waterproof mascara, lipstick), shampoos and conditioners, and pressure sensitive adhesives [7]. Even though the first publication regarding MQ resins was published in 1946 [8], their molecular structures were mostly absent in the literature until the seminal review by Flagg and McCarthy [3]. This is largely because the production methods for MQ resins lead to complex mixtures both of structures and molecular weights [9]. As a consequence, it is very difficult to establish structure/property relationships for these highly reticulated resins. An excellent recent review of silicone resins is provided in [10].

As noted above, most syntheses of MQ and related resins involve acid and/or base catalysis, conditions under which siloxane bonds undergo metathesis (redistribution) and other (degradative) reactions. The objective of this work is to better understand how the 3D structure of silicone oils affects the performance of oils in adhesive silicone gels. Therefore, a library of well-defined silicone oils was prepared using the Piers-Rubinsztajn reaction, which allows the preparation of precise, highly branched structures without redistribution starting from hydrosilanes and an alkoxysilane or silanol in the presence of B(C_6_F_5_)_3_ (Figure 1D) [11,12]. The reaction is orthogonal to platinum-catalyzed hydrosilylation (Figure 1E) [13], which was additionally used. Using these two reactions, the rapid preparation was achieved of well-defined 3D silicone resins (MDTQ resins) with different degrees of branching through incorporation at different levels of both crosslinking trifunctional T (MeSiO_3/2_) and tetrafunctional Q (SiO_4/2_) groups, and various degrees of difunctional chain extender D groups (MeSiO_2/2_) and terminal M groups (Me_3_SiO). The structure/property relationships with respect to viscosity of the oils themselves and the ability of the oils to affect tack in silicone adhesives as a function of branching were investigated.

## 2. Results

### 2.1. Synthesis of a Library of MDTQ Resins

**Figure 2:**
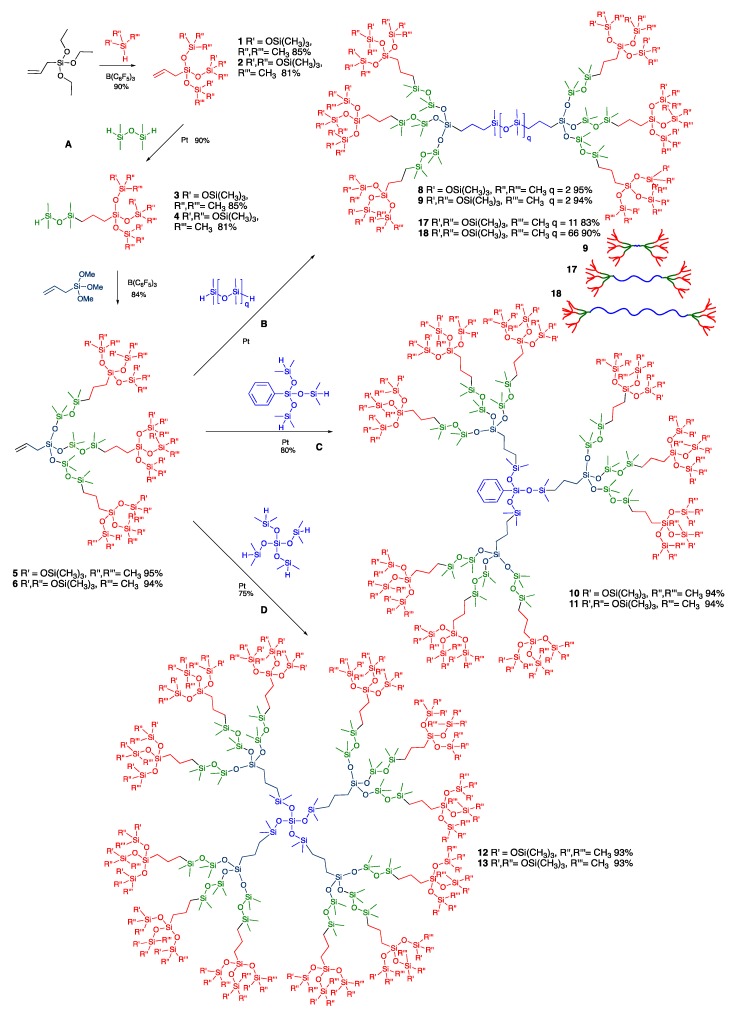
Synthesis of branched silicones starting from dendrons.

**Figure 3:**
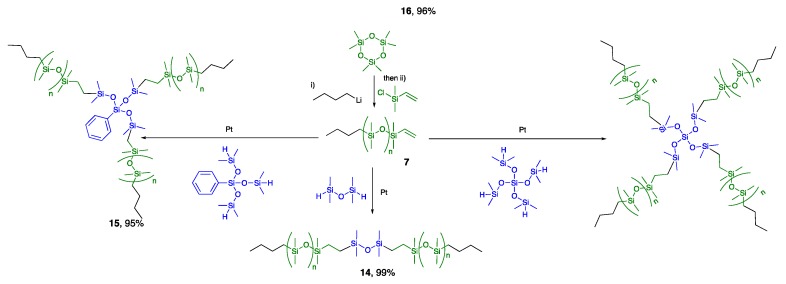
Synthesis of branched silicones starting from linear materials.

Monoallyl-terminated silicone dendrons with different degrees of branching were prepared as shown in Figure 2A [14]. First, allyltriethoxysilane underwent the Piers-Rubinsztajn (PR) reaction with either pentamethyldisiloxane (**T(DM)_3_** series) or 1,1,1,1,3,5,5,5-heptamethyltrisiloxane (**T(TM_2_)_3_** series) to afford compounds **1** or **2** in good yield. The products were subjected to Pt-catalyzed hydrosilylation with a stoichiometric excess of 1,1,3,3-tetramethyldisiloxane to produce compound **3** and **4**, respectively, bearing a single Si-H functional group. The iterative process was repeated with allyltrimethoxysilane to generate allyl-terminated silicone dendrons **5** and **6** in excellent yield. For purposes of comparison, a mono-vinyl terminated linear silicone **7** of similar molecular weight was prepared in a ring-opening polymerization using *n*-BuLi as initiator to open the strained cyclic monomer D_3_ ((Me_2_SiO)_3_) ring, followed by capping with chlorodimethylvinylsilane as shown in Figure 3.

Libraries of branched silicones resins with different branching densities were prepared in a convergent approach using these three moieties. Simple and efficient platinum-catalyzed hydrosilylation led to 3D silicone resins with cores having 2-fold symmetry (1,1,3,3-tetramethyldisiloxane) **8**, **9**, 3-fold symmetry (tris(dimethylsiloxy)phenylsilane) **10**, **11**, and 4-fold symmetry (tetrakis(dimethylsiloxy)silane) **12**, **13**, respectively (Figure 2B–D). Mono-vinyl terminated linear chains were subjected to the same hydrosilylation reactions to produce linear **14** (2-fold) and branched (3-fold **15**, and 4-fold **16** symmetry) silicone oils (Figure 3). The structures of the silicone dendrimers were confirmed by ^1^H NMR and high-resolution ESI (electrospray ionization) mass spectrometry or MALDI (Matrix Assisted Laser Desorption/Ionization) to ensure the absence of defects; characterization of these compounds by NMR was particularly facile because of their high symmetry. It should be noted that the major hydrosilylation isomer was the 1,2-product formed via (SiCH_2_CH_2_Si, Figure 1E and Figure 2), however, the formation of small amounts of the 1,1-isomer (∼10% SiCHCH_3_Si) was also observed in the ^1^H NMR. These results are in line with the gel permeation chromatography (GPC) data, which exhibited a small shoulder peak on the high molecular weight side of the curve, as has previously be noted by us [11].

The design and synthesis of the abovementioned 9 compounds permitted an investigation of structure and properties relationships of the MDTQ resins with respect to both branching density—linear **14**, **15**, **16**; medium branching **8**, **10**, **12** (**T(DM)_3_** series); to highly branching **9**, **11**, **13** (**T(TM_2_)_3_** series)—and symmetry: 2-fold v 3-fold v 4-fold. One additional structural motif was examined. Additional two-fold symmetry compounds of the **T(TM_2_)_3_** series were prepared with different spacer lengths using 1,1,3,3-tetramethyldisiloxane, DMS-H11, or DMS-H21 to afford compounds **9**, **17**, and **18** in good yield (Figure 2). In this latter series of compounds, the branching dendrons remained identical, but the fraction of D units between dendrons was increased, which allowed the investigation of the role D units play in modifying the viscoelastic behavior of silicone elastomers.

### 2.2. Viscosity

**Figure 4:**
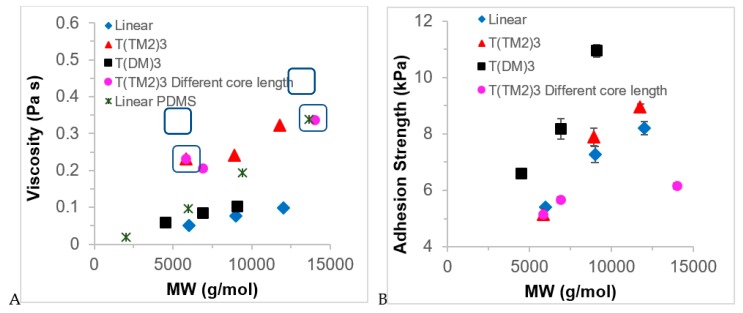
(**A**) Viscosity plotted a function of branching type and molecular weight. Samples in boxes have the same fraction of M groups. (**B**) The influence of MW on the adhesion work of oil filled elastomers with 25 wt% loading. Note the small change in viscosity between the 2-fold hyperbranched polymer ▲ **9** M_n_ = 5860 g mol^−1^ and the analogue with a ~8000 g mol^−1^ spacer between the same dendrons ● **18** 14,000 g mol^−1^. The error bars represent the standard deviation of 3 measurements but were so small that they fall underneath the marker (the data may be found in Table S2).

The effect of branching density on the viscosity of the silicone oils alone was measured using rheology (Figure 4A, Table S2). It is apparent that molecular weight of the oil is a minor player in determining viscosity; branching plays a much more important role. At a given molecular weight (MW), the viscosities of the linear polymers were slightly lower than those of the lightly branched polymers (**T(DM)_3_** series) of comparable molecular weight. However, the more densely branched dendrimers (**T(TM_2_)_3_** series) exhibited much higher viscosities. Increasing the spacing between dendrimers on a linear silicone chain (**9** vs. **17**, **18**) had little additional effect on viscosity; that is, with respect to viscosity, the D units (Me_2_SiO) have only a small impact when compared to T and, likely, Q units.

### 2.3. Modulus and Work of Adhesion

**Figure 5:**
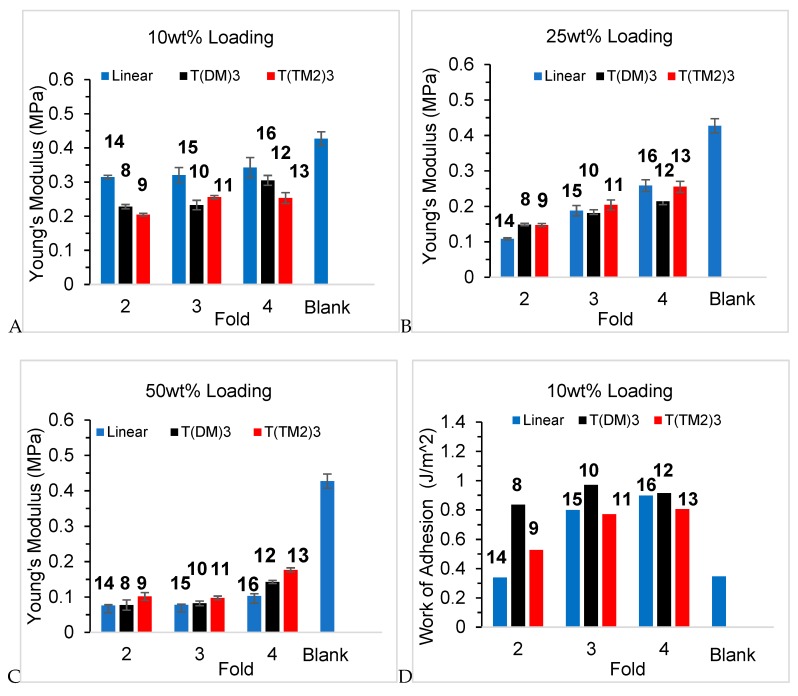

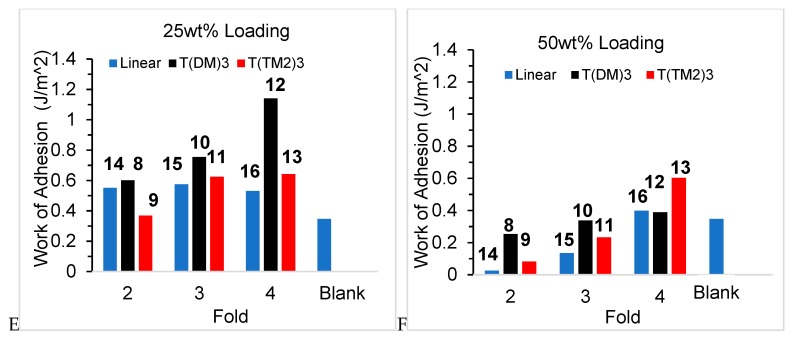
(**A**–**C**) Young’s modulus of silicone oils 8–16 at three weight fractions (10, 25, 50%) in the control silicone elastomer. (**D**–**F**) Adhesion work as a function of loading (10, 25, 50%) of oil in the elastomer.

Adding the branched oils **8**–**16** to a silicone pre-elastomer formulation **19** and then curing converted the materials into gels. Unsurprisingly, the higher the loading of low MW oils (<12,000 g mol^−1^), the softer were the materials (Young’s modulus, Figure 5A–C, Table S4). In general, at a given loading, the differences in modulus were subtle. However, one trend that was matched in almost all the series was that more highly branched oils–particularly the 4-armed branches–exhibited higher degrees of reinforcement, or lower degree of plasticization of the network, depending on one’s point of view. While it is tantalizing to associate the nature of the branching with these changes, there is a concomitant change in molecular weight with higher branching (Figure 5A–C, Table S4), which may be of equal or more importance in the changes in hardness.

One of the objectives of this work was to make materials that had potential utility as tack promoters and, therefore, the tack strength of the MTDQ resin-filled silicone elastomers were measured (Table S3). A silicone elastomer was prepared, using platinum-catalyzed hydrosilylation, from vinyl-terminated linear silicones and a pendent HSi-containing polymers (and no additional fillers) in the presence of the **8**–**16** oils at 3 loading levels, 10, 25 and 50 wt%. The work of adhesion is summarized in Table 1. Two general trends were observed: work of adhesion decreased for all series as the loading of oil in the gel was increased; and, the 4-armed oils performed more effectively than 3 or 2-armed oils at a given loading (Figure 5D–F). In addition, at 25% loading, the **T(DM)_3_** series **8**, **10**, **12** performed better than either the linear branched materials **14**–**16** or the **T(TM_2_)_3_** series **9**, **11**, **13**.

### 2.4. Optical Microscopy and Transmittance

**Figure 6:**
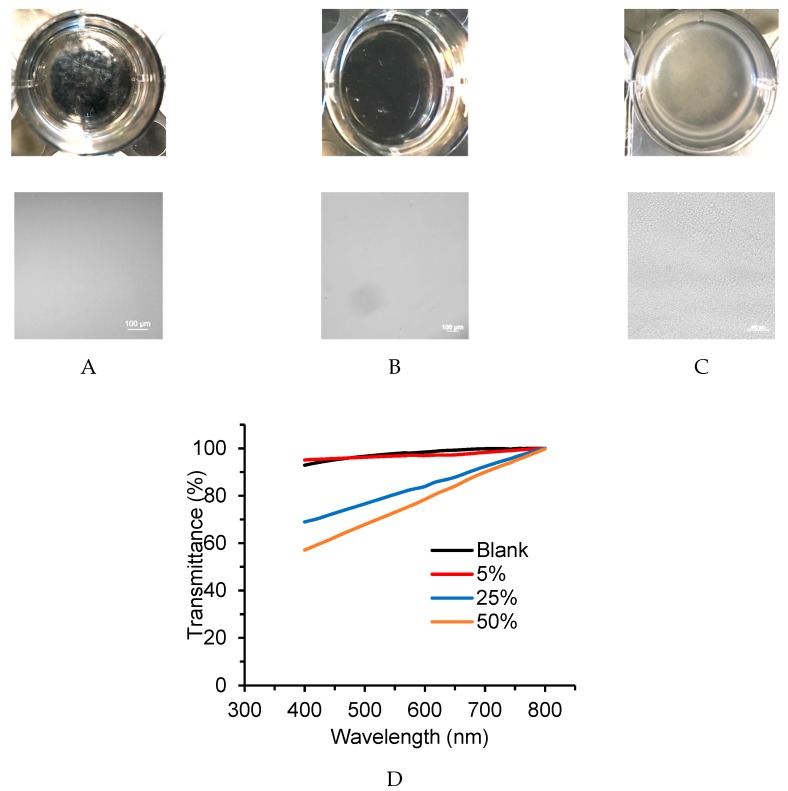
Optical pictures of samples containing 50 wt% different oils in cured silicone gels (Top optical image, bottom microscope image X10) (**A**) linear polydimethylsiloxane MW 28,000 g mol^−1^, (**B**) 4 arm linear **16**, (**C**) 4 arm **T(TM_2_) 13**. (**D**) Transmittance at various wavelengths of different concentrations of **13** the uncured silicone pre-elastomer.

Silicone oils of different constitution are typically infinitely miscible in other silicone oils. It was, therefore, surprising to note that, unlike all other oils, a cloudy dispersion formed when the 4 arm **(TM_2_)_3_** hyperbranched polymer **13** was added to the pre-elastomer (Figure 6A–C); transmittance decreased with the increasing loading of **13** to the pre-elastomer. These changes were not a consequence of the higher MW of 4 arm **13** (MW 11,776 g mol^−1^); 28,000 MW linear silicone did not phase separate in the pre-elastomer at the same loading. The degree to which phase separation occurred was concentration dependent, as shown from transmittance data (Figure 6D). The immiscibility is related to the lower work of adhesion for these samples, as is addressed below.

## 3. Discussion

### 3.1. Viscosity

All of the oils prepared have rather low molecular weight. As is the case for commercial linear polydimethylsiloxane, it is apparent for these oils that the molecular weight of the oil is a minor player in viscosity; branching plays a much more important role (Figure 4). Viscosities of the 2-, 3- and 4- branched linear polymers were just lower than those of the lightly branched **T(DM)_3_** series **8**, **10**, **12** of comparable molecular weight. However, the more densely branched dendrimers of the **T(TM_2_)_3_** series **9**, **11**, **13** exhibited much higher viscosities. Spacing the hyperbranched units on a linear silicone chain **9** vs. **17**, **18**–had little additional effect on viscosity; the D units (Me_2_SiO) play only a small part here.

The molecular weight dependence of viscosity for the linear polymers is well reported in the literature. The viscometric behavior can be divided into two distinct regions, below and above the critical molar mass for entanglement, which for linear silicone oils is MW_ENT_∼29,000 g mol^−1^ [15]. This molar mass represents the point at which the sample begins to show the presence of a temporary entanglement network structure and sharp viscosity increase is normally observed [16].

All the oils examined in this report are well below the entanglement limit. The dramatic increase of viscosity for the more branched **T(TM_2_)_3_** series (**9**, **11**, **13**, **17**, **18**) could have two origins; entanglement or interparticle friction. Malkin [17] looked at the viscosity and viscoelasticity behavior of (internally crosslinked) polymethylsilsesquioxane nanoparticles within polydimethylsiloxanes. A rapid increase in viscosity was reported as low MW polymethylsilsesquioxanes were added. They considered that the entanglement mechanism is not likely to be important for their crosslinked particles in polydimethylsiloxanes because they do not possess long chains and therefore ascribe the viscosity increase to frictional forces arising from aggregation/deaggregation processes of the particles. The **T(TM_2_)_3_** series oils are not crosslinked. While entanglement in the hyperbranched polymers under consideration should more likely occur than with crosslinked particles, it cannot be a dominant mechanism. Otherwise, the viscosity would be expected to be significantly exacerbated when the overall molecular weight increases. A comparison of the 2- fold symmetry compounds **9** vs. **18** shows essentially the same viscosity regardless of the addition of a 8000 g mol^−1^ chain (inside the boxes Figure 4A, **9** ▲ M_n_ = 5860 g mol^−1^ vs. **18** ● 14,000 g mol^−1^).

### 3.2. Interactions between the Oils and the Network: Modulus, Work of Adhesion and Phase Separation

Adding oils to the silicone elastomers converts the materials into gels. Unsurprisingly, the higher the loading of low MW oils (<12,000 g mol^−1^), the softer were the materials (Young’s modulus, Figure 5A–C, Table S4). In general, at a given loading, the differences in modulus were subtle. However, one trend that was found in almost all the series was that more highly branched oils–particularly the 4-armed branches–exhibited higher degrees of reinforcement, or lower degree of plasticization of the network, depending on one’s point of view. While it is tantalizing to associate branching with these changes, there is a concomitant change in molecular weight with higher branching (Table S4), which may be of equal or more importance in the changes in hardness.

Several features must be considered when analyzing the tack data: quantity of oil loaded in the gel, type of branch, symmetry of the molecule, and overall molecular weight of the oil. There is a nearly linear reduction in tack strength for a given oil as its composition in the gel is increased from 0–50 wt% (Table S3); changes in Young’s moduli followed the same trend (Figure 5A–C). This may be due to the elastic deformation caused by the large differences in hardness [18]. When the effects of the Young’s modulus were normalized by calculating the work of adhesion, samples with 10–25 wt% loading show better adhesion property than the blank sample (Figure 5D–F). Once loading of the oils was increased to 50 wt% the resulting gels exhibited adhesion comparable to the parent elastomer, which suggests that the hyperbranched polymers are acting as plasticizers and/or migrate onto the elastomer surface where they lubricate the interaction betweens the probe and the gel. There is insufficient data to establish the relative importance of 4- versus 3- or 2- armed analogues vs. the effects of MW (Figure 5D–F, Table 1). However, a comparison the work of adhesion in gels with 25 wt% loading makes clear that 3D structure plays a larger role in adhesion work than molecular weight. Oils comprised of linear or **T(TM_2_)_3_**-type structures had very similar work of adhesion, compare **9** v **14**, **11** v **15**, **13** v **16**, (Figure 4B, Table 1) at a given MW, while the **T(DM)_3_**-derived structures exhibited much higher work of adhesion even at much lower MW. Two factors can be ascribed to this behavior: interaction of the oils with the network—a type of entanglement, and solubility of the oil in the elastomer. Oils of the **T(DM)_3_** series will interact more efficiently with the (D-rich) elastomer network than analogous 2-, 3-, 4-fold polymers with linear chains. That is, the increased viscosity due to interaction of the oils themselves manifests when present in the gel. During the tack measurement a lower fraction of the force is stored in the elastic network when more viscous, soluble oils are present [19]. On this basis, gels containing the **T(DM)_3_** series oils should have even higher tack, but solubility of the oil in the network must also be considered.

Gels containing the **T(DM)_3_** series oil **13** were opaque–the M-rich oil did not dissolve in the D-rich elastomer (Figure 6). While opacity was not obvious, the M-rich lower MW analogues **9**, **11** are similarly expected to be less soluble within the gels. The ability of a gel to provide tack depends on the solubility of the oils that swell the elastomer. In a key study, Lenhart and Cole compared tack adhesion of gels containing soluble and less soluble oils [20]. They demonstrated significantly lower tack for the gels containing less soluble oils. This observation is also consistent with the self-association behavior of hyperbranched polymers reported in the literature. Enders and Langenbach used their study to elucidate the thermodynamic explanations for the demixing of hyperbranched polymer [21]. They used lattice cluster theory to calculate the phase diagram for the dendrimeric polymer, which predicted large demixing regions for mixtures of linear polymers and hyperbranched polymers. It was concluded that an increase of the molecular weight (and for dendrimers, generation number) leads to a larger miscibility gap. As a consequence, self-association between hyperbranched polymers led to phase separation. Mavrič et al. [22,23] similarly found that dendrimeric polysilanes agglomerate at room temperature. The dendrimers maintain the size scaling typical for globular polymers in all temperature and concentration regimes and the strong, attractive van der Waals facilitate ‘self’ interactions between the polymers [24]. That is, the mixture stays in the two-phase region of the Flory-Huggins phase diagram, leading to miscibility problems, aggregate formation and subsequent precipitation.

**Figure 7:**
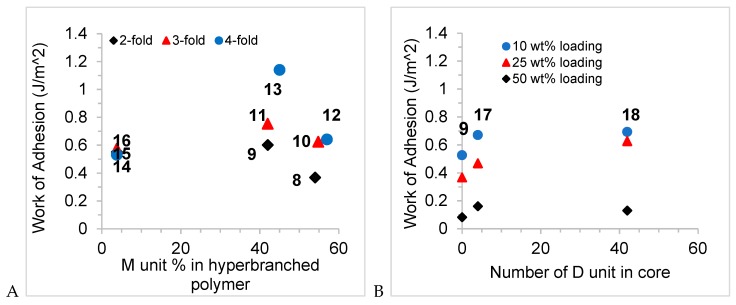
(**A**) Adhesion work to gels (25 wt% loading) as a function of M unit mol% on the hyperbranched oils). (**B**) Adhesion work as a function of different core length in the elastomer.

MQ resins used as reinforcing agents in industry are typically between MW 1000–10,000 g mol^−1^. As noted in the excellent review by Flagg and McCarthy [3], little is known about the specific structural features of these compounds and how they specifically interact within an elastomer or gel. The model compounds **8**–**18**, which fall within this molecular weight range, shed some light on the characteristics that can benefit gel performance. Unlike MQ and related resins, none of the synthesized hyperbranched polymers are highly crosslinked; they possess low fractions of T and Q monomer units. D units plays a minor role in the viscosity exhibited by the oils and, when incorporated into gels, are not very effective at improving tack; the linear series were much less effective than the **T(DM)_3_** series that can more effectively act with the network to resist external forces. Adding a long D chain between branched materials led essentially to no changes in tack (Figure 4B). However, an overabundance of M groups in the **T(TM_2_)_3_** series leads to phase separation from the network leading to plasticization, rather than reinforcement. These outcomes are readily seen in the plots of adhesion vs. M% and D unit is the core (Figure 7). Thy suggest that more effective MDTQ resins for tack maybe designed by incorporating increased branching, by using lower fractions of D units, but not to the extent that the oils become sufficiently M-rich that they undergo phase separation.

Branched silicone oils of precise structure provide guidance for the parameters needed to improve adhesion of gels. Several factors were observed to play a role: molecular weight, symmetry of the oil, branch density and loading of the oil in the gel. None of these factors is individually predictive for the impact of adhesion. However, with these simple empirical rules for structure/property relationships it becomes possible to design oils that will provide the desired physical properties in silicone gels.

## 4. Materials and Methods

### 4.1. Starting Materials

Allyltrimethoxysilane, pentamethyldisiloxane 1,1,1,1,3,5,5,5-heptamethyltrisiloxane, tetramethyldisiloxane, hexamethylcyclotrisiloxane (D_3_), and H-terminal dimethylsilicone (H-PMDS-H, HMe_2_Si(OSiMe_2_)_n_SiMe_2_H, DMS-H11 ~1000 g mol^−1^; DMS-H21, MW ~5000 g mol^−1^) were purchased from Gelest and used as received. Tris(dimethylsiloxy)phenylsilane, tetrakis(dimethylsilyl)-orthosilicate, chlorodimethylvinylsilane, and *n*-butyllithium solution (2.5 M) in hexanes were purchased from Sigma Aldrich and used as received. Tris(pentafluorophenyl)borane (B(C_6_F_5_)_3_, BCF catalyst) was purchased from Alpha Aesar and used as received. Commercial solvents: hexane, dichloromethane and toluene were dried over activated alumina prior to use.

### 4.2. Characterization Methods

^1^H NMR and ^13^C NMR experiments were recorded at room temperature and performed on a Bruker Avance 600 MHz nuclear magnetic resonance spectrometer. Note: in the ^1^H NMR of compounds formed by hydrosilylation, only the data for the major, terminal hydrosilylation isomer (SiCH_2_CH_2_Si) are reported, which comprises >90% of the product. The minor peaks from the other isomer (SiMeCHSi) were only identifiable in low generation molecules.

High-resolution mass Spectrometry was performed with a Hi-Res Waters/Micromass Quattro Global Ultima (Q-TOF mass spectrometer (MS)). MALDI mass spec was collected on a Bruker Autoflex III at McGill University by Dr Nadim Saade with dithranol as the matrix used for most of the samples. ESI-MS Instrument: Exactive Plus Orbitrap (Thermo Scientific, Waltham, Massachusetts). Sample concentration used were~50–100 μM.

GPC data was collected on a Viscotek GPC Max (VE 2001 GPC Solvent/Sample Module) using a Viscotek VE 3580 RI Detector and a Viscotek 270 Dual Detector using a PolyAnalytik SupeRes PAS-101 (8 mm × 30 cm) column with a hard styrene-divinylbenzene gel; single pore, 6nm particle size, a plate count >18,000, and an exclusion limit of 1.5K. The samples were run in toluene. Polystyrene was used as standard for GPC calibration.

Young’s modulus data was collected using a MACH-1 micromechanical testing instrument equipped with a hemispherical indenter (diameter:0.5 mm). The results were analyzed by the add-on software Mach-1 Analysis. The analysis model (Elastic model in indention) for finding the Young’s modulus from an indentation test was provided by the vender (Biomomentum), which was developed based on the mathematical model established by Hayes et al. [25]. The elastomers in 12-well plate (thickness: 5 mm) were measured in triplicate, with error bars representing the standard deviation of the replicate measurements.

The adhesion strength of elastomeric materials was measured using a MACH-1 micromechanical testing instrument equipped with a punch (diameter:13mm) following the method and optimized conditions described in the literature [26,27]. Briefly, the elastomers, formed in a 12-well plate, were mounted onto the stage of the instrument. The steel punch moved down to come in contact with the elastomer to be tested. When the contact stress reached 1.5 N, the punch was programed to hold at that position for 3 min to establish sufficient contact. The probe was then pulled away at a de-bonding velocity of 1 mm s^−1^. The displacement of the punch and the force applied on it was collected by the instrument. The results are shown in Table S3.

To normalize the influence of Young’s modulus on the tack strength, work of adhesion was calculated with Young’s modulus and tack strength using the following equation (Table 1, Table S4).

(1) $$W=\frac{a\pi P^{2}}{10E}$$

where W (J/m^2^) is the work of adhesion; a is the radius of the punch (6.5 × 10^−3^ m); P is the tack strength measured (Pa); E is the Young’s modulus of the samples. Viscosity measurements were carried out on a TA Instruments Discovery HR-2 Hybrid Rheometer with a 40 mm steel Peltier plate. The shear rate was set between 0 and 500 s^−1^ (Table S2).

### 4.3. Synthesis of All Compounds

#### 4.3.1. Synthesis of **1**

Allyltrimethoxysilane (5.0328 g, 31.017 mmol) was added to a 500 mL oven-dried round-bottomed flask. Dry hexanes (~10 mL) were added to the reaction and the flask was capped and flushed with nitrogen. BCF catalyst solution (110 μL, 0.0365 g/mL toluene, 7.84 × 10^−3^ mmol) was added before slowly adding in excess pentamethyldisiloxane (22.7582 g, 153.401 mmol). After 2 h the reaction was shown to be complete by ^1^H NMR and ~1.5 g of neutral alumina was added to the flask to remove the BCF. The solution was left to stir for ~1 h before filtering the product through Celite and rinsing with hexane. Hexanes were removed under reduced pressure, yielding 14.6443 g of **1** (85% yield). ^1^H NMR (CDCl_3_, 600 MHz): δ 6.13 (dd, 1H, *J* = 14.8, 20.4 Hz), 5.93 (dd, 1H, *J* = 3.8, 14.8 Hz), 5.74 (dd, 1H, *J* = 3.8, 20.4 Hz), 1.57 (d, 2H, *J* = 7.9 Hz), 0.19–0.05 (m, 46H) ppm

#### 4.3.2. Synthesis of **2**

Allyltrimethoxysilane (5.0087 g, 30.868 mmol) was added to a 500 mL oven-dried round-bottomed flask. Dry hexanes (~10 mL) were added to the reaction and the flask was capped and flushed with nitrogen. BCF catalyst solution (600 μL, 0.0400 g/mL toluene, 4.68 × 10^−2^ mmol) was added before slowly adding excess bis(trimethylsiloxy)methylsilane (34.4652 g, 154.900 mmol). The reaction was kept stirring for 3 h, and then neutral alumina (~1 g) was added to the flask. The solution was left to stir for ~1 h before filtering the product through Celite and rinsing with hexane. Hexanes were removed under reduced pressure. Excess bis(trimethylsiloxy)methylsilane was removed by distillation at 120 °C for ~45 min yielding 19.4302 g of **2** (81% yield). ^1^H NMR (CDCl_3_, 600 MHz): δ 6.13 (dd, 1H, *J* = 14.8, 20.4 Hz), 5.93 (dd, 1H, *J* = 3.8, 14.8 Hz), 5.74 (dd, 1H, *J* = 3.8, 20.4 Hz), 1.57 (d, 2H, *J* = 7.9 Hz), 0.20–0.02 (m, 70H) ppm.

#### 4.3.3. Synthesis of **3**

In an oven-dried round bottomed flask, **1** (5 g, 8.94 mmol) was added to a solution of tetramethyldisiloxane (6 g, 44.7 mmol) in 15 mL of dry hexane. A drop of platinum (0) 1,3-divinyl-1,1,3,3-tetramethyldisiloxane catalyst (10 μl of a 2% by wt in xylenes solution, 2.24 × 10^−2^ mmol) was added. The solution was treated with activated charcoal (∼1 g) and the mixture allowed to stir for 1 h. The solution was then filtered and concentrated in vacuo. The residue was then heated to 100 °C under high vacuum (1 mmHg) to ensure complete removal of excess starting materials, yielding colorless liquid **3** (4.95 g, 80% yield). ^1^H NMR (CDCl_3_, 600 MHz): δ 4.70 (m, 1H,) 1.45 (m, 2H), 0.61 (m, 4H), 0.21–0.02 (m, 57H) ppm. High resolution mass spectrometry (ES (electrospray) Positive mode): *m*/*z* [M + NH_4_]^+^ calc. = 710.2914; found = 710.2909.

#### 4.3.4. Synthesis of **4**

In an oven-dried round bottom flask, **2** (10 g, 12.7 mmol) was added to a solution of tetramethyldisiloxane (8.59 g, 63.9 mmol) in 20 mL of dry hexane. A drop of platinum (0) 1,3-divinyl-1,1,3,3-tetramethyldisiloxane catalyst (10 μl of a 2 wt% in xylenes solution, 2.24 × 10^−2^ mmol) was added. The solution was stirred at room temperature under a nitrogen atmosphere for 3 h. The solution was treated with activated charcoal (∼1 g) and the mixture allowed to stir for 1 h. The solution was then filtered and concentrated in vacuo. The residue was then heated to 100 °C under high vacuum (1 mmHg) to ensure complete removal of excess starting materials, yielding colorless liquid **4** (10.35 g, 89% yield). ^1^H NMR (CDCl_3_, 600MHz): δ 4.60 (m, 1H,) 1.45 (m, 2H), 0.63 (m, 4H), 0.17–0.06 (m, 75H) ppm. High resolution mass spectrometry High resolution mass spectrometry (ES Positive mode): *m*/*z* [M]^+^ calc. = 915.3251; found = 915.3218.

#### 4.3.5. Synthesis of **5**

Allyltrimethoxysilane (0.622 g, 3.83 mmol) was added to a 500 mL oven-dried round-bottomed flask. Dry hexanes (~10 mL) were added to the reaction and the flask was capped and flushed with nitrogen. BCF catalyst solution (140 μL, 0.025 g/mL toluene, 6.83 × 10^−3^ mmol) was added before slowly adding **3** (8 g, 11.5 mmol). After 2 h the reaction was shown to be complete by ^1^H NMR and ~1.5 g of neutral alumina was added to the flask to remove the BCF. The solution was left to stir for ~1 h before filtering the product through Celite and rinsing with hexane. Hexanes were removed under reduced pressure. The residue was subjected to kugelrohr distillation at 170 °C to remove impurities, yielding 7.09 g of **5** (84% yield). ^1^H NMR (CDCl_3_, 600 MHz): δ 5.78 (m, 1H), 4.94–4.90 (m, 1H), 4.88–4.85 (m, 1H), 1.54–1.52 (m, 2H), 1.41–1.39 (m, 6H), 0.64–0.57 (m, 12H), 0.10–0.04 (m, 171H) ppm. High resolution mass spectrometry (ES Positive mode): *m*/*z* [M + Na]^+^ calc. = 2213.7391; found = 2213.7465.

#### 4.3.6. Synthesis of **6**

Allyltrimethoxysilane (0.177 g, 1.09 mmol) was added to a 250 mL oven-dried round-bottomed flask. Dry hexanes (~10 mL) were added to the reaction and the flask was capped and flushed with nitrogen. BCF catalyst solution (13 μL, 0.025 g/mL toluene, 0.63 × 10^−3^ mmol) was added before slowly adding **4** (3 g, 3.27 mmol). After 2 h the reaction was shown to be complete by ^1^H NMR and ~1.5 g of neutral alumina was added to the flask to remove the BCF. The solution was left to stir for ~1 h before filtering the product through Celite and rinsing with hexane. Hexanes were removed under reduced pressure. The residue was subjected to kugelrohr distillation at 170 ^°^C to remove impurities, yielding 2.53 g of **6** (82% yield). ^1^H NMR (CDCl_3_, 600 MHz): δ 5.83–5.75 (m, 1H), 4.9–4.90 (m, 1H), 4.8–4.85 (m, 1H), 1.5–1.52 (m, 2H), 1.4–1.40 (m, 6H), 0.6–0.60 (m, 12H), 0.20–0.01 (m, 225H) ppm. High resolution mass spectrometry (ES Positive mode): *m*/*z* [M + Na]^+^ calc. = 2880.9161; found = 2880.9275.

#### 4.3.7. General Procedure for Synthesis of **8**–**13** (Shown for **9**)

In an oven-dried round bottom flask, **6** (4.836 g, 1.69 mmol) was added to a solution of tetramethyldisiloxane (0.144 g, 0.84 mmol) in 5 mL of dry hexane. A drop of platinum (0) 1,3-divinyl-1,1,3,3-tetramethyldisiloxane catalyst (10 μl of a 2 wt% in xylenes solution, 2.24 × 10^−2^ mmol) was added. The solution was stirred at room temperature under a nitrogen atmosphere for 3 h. The solution was treated with activated charcoal (∼1 g) and the mixture allowed to stir for 1 h. The solution was then filtered and concentrated in vacuo. The residue was subjected to Kugelrohr distillation at 170 °C to remove impurities, yielding colorless liquid **9** (4.46 g, 88% yield). ^1^H NMR (CDCl_3_, 600 MHz): δ 1.43–1.38 (m, 16H), 0.64–0.56 (m, 32H), 0.14–0.01 (m, 462H) ppm. High resolution mass spectrometry (ES Positive mode): *m*/*z* [M + Na]^2+^ calc. = 2943.9518; found = 2943.9576.

**^1^H NMR and HR-MS for 8 (Yield: 95%):**^1^H NMR (CDCl_3_, 600 MHz): δ 1.45–1.38 (m, 16H,), 0.65–0.55 (m, 32H), 0.11–0.01 (m, 354H) ppm. HR-MS (ES Positive mode): *m*/*z* [M + Na]^+^ calc. = 4538.5774; found = 4538.5563.

**^1^H NMR and HR-MS for 10 (Yield: 94%):**^1^H NMR (CDCl_3_, 600 MHz): δ 7.50–7.48 (m, 2H), 7.34–7.31 (m, 1H), 7.29–7.26 (m, 2), 1.43–1.38 (m, 24H,), 0.64–0.56 (m, 48H), 0.14–0.01 (m, 532H) ppm. High resolution mass spectrometry (ES Positive mode): *m*/*z* [M + Na]^2+^ calc. = 3475.1757; found = 3475.1699

**^1^H NMR and HR-MALDI for 11 (Yield: 94%):**^1^H NMR (CDCl_3_, 600 MHz): δ 7.50–7.48 (m, 2H), 7.34–7.31 (m, 1H), 7.29–7.26 (m, 2), 1.43–1.38 (m, 24H,), 0.64–0.56 (m, 48H), 0.14–0.01 (m, 694H) ppm. MALDI: *m*/*z* [M]^+^ calc.= 8900.853, found = 8900.311

**^1^H NMR and HR-MALDI for 12 (Yield: 93%):**^1^H NMR (CDCl_3_, 600 MHz): δ 1.42–1.35 (m, 32H), 0.63–0.53 (m, 64H), 0.08–0.01 (m, 708H) ppm. MALDI: *m*/*z* [M]^+^ calc.= 9091.083, found = 9091.182

**^1^H NMR and HR-MALDI for 13 (Yield: 92%):**^1^H NMR (CDCl_3_, 600 MHz): δ 1.42–1.35 (m, 32H), 0.63–0.53 (m, 64H), 0.09–0.01 (m, 924H) ppm. MALDI: *m*/*z* [M]^+^ calc.= 11,755.759, found = 11,755.567.

#### 4.3.8. General Procedure for Synthesis of **14**–**16** (Shown for **14**)

Mono-vinyl terminated PDMS **7** was prepared according to the literature procedure [14]. The Mn of this polymer was 3165 g mol^−1^ and dispersity (*Đ*_M_) was 1.2. In an oven-dried round bottom flask, mono-vinyl-terminated PDMS (4 g, 1.27 mmol) was added to a solution of tetramethyldisiloxane (0.085g, 0.632 mmol) in 5 mL of dry hexane. The ^1^H NMR experiment for this reaction mixture was carried out to make sure that the stoichiometry ratio between Si-H and vinyl group was exactly 1:1. Then, a drop of platinum (0) 1,3- divinyl-1,1,3,3-tetramethyldisiloxane catalyst (10 μl of a 2% by wt in xylenes solution, 2.24 × 10^−2^ mmol) was added. The solution was stirred at room temperature under a nitrogen atmosphere for 3 h. The solution was treated with activated charcoal (∼1 g) and the mixture allowed to stir for 1 h. The solution was then filtered and concentrated in vacuo. The residue was subjected to Kugelrohr distillation at 170 °C to remove impurities, yielding colorless liquid **14** (3.47 g, 85% yield). ^1^H NMR (CDCl_3_, 600 MHz): δ 1.26–1.21 (m, 8H) 0.80 (t, 6H, *J* = 6.8 Hz), 0.48 (t, 4H, *J* = 6.7 Hz) 0.37–0.32 (m, 8H)) 0.09–0.04 (m, 510H) ppm. GPC: Mn: 7330, Mw: 7960, and PDI: 1.08.

**^1^H NMR and GPC for 15 (Yield: 89%):**^1^H NMR (CDCl_3_, 600 MHz): δ 7.50–7.48 (m, 2H), 7.34–7.31 (m, 1H), 7.29–7.26 (m, 2), 1.35–1.29 (m, 12H) 0.93–0.86 (m, 9H), 0.55–0.53 (m, 6H), 0.47–0.39 (m, 12H) 0.37–0.32 (m, 8H),0.09–0.04 (m, 852H). GPC: M_n_: 10835, M_w_: 11745, and PDI: 1.08.

**^1^H NMR and GPC for 16 (Yield: 90%):**^1^H NMR (CDCl_3_, 600 MHz): δ 1.26–1.21 (m, 16H) 0.80 (m, 12H), 0.48 (m, 8H) 0.37–0.32 (m, 16H), 0.09–0.04 (m, 1120H) ppm. GPC: M_n_: 13,105, M_w_: 16,250, and PDI: 1.24.

#### 4.3.9. General Procedure for Synthesis of **17**–**18** (Shown for **17**)

In an oven-dried round bottom flask, **5** (4 g, 1.39 mmol) was added to a solution of DMS-H11 from Gelest (0.823 g, 0.698 mmol) in 5 mL of dry hexane. The ^1^H NMR experiment for this reaction mixture was carried out to make sure that the stoichiometry ratio between Si-H and vinyl group was exactly 1:1. Then, a drop of platinum (0) 1,3- divinyl-1,1,3,3-tetramethyldisiloxane catalyst (10 μL of a 2 wt% in xylenes solution, 2.24 × 10^−2^ mmol) was added. The solution was stirred at room temperature under a nitrogen atmosphere for 3 h. The solution was treated with activated charcoal (∼1 g) and the mixture allowed to stir for 1 h. The solution was then filtered and concentrated in vacuo. The residue was subjected to kugelrohr distillation at 170 °C to remove impurities, yielding colorless liquid **17** (4.08 g, 83% yield). ^1^H NMR (CDCl_3_, 600 MHz): δ 1.42–1.35 (m, 10H), 0.63–0.53 (m, 20H), 0.09–0.01 (m, 568H) ppm. GPC: Mn:7160, Mw: 9590, PDI:1.33.

**^1^H NMR and HR-MALDI for 18 (Yield: 90%):**^1^H NMR (CDCl_3_, 600 MHz): δ 1.42–1.35 (m, 10H,), 0.63–0.53 (m, 20H), 0.09–0.01 (m, 1046H) ppm. GPC: Mn:14,000, Mw: 19,895, PDI:1.42.

#### 4.3.10. Preparation of MTDQ Resin/Filler Silicone Elastomers

A master batch of hydrosilylation cure base was made by mixing vinyl-terminated silicone DMS V31 (50.0 g, H_2_C=CH-SiMe_2_(OSiMe_2_)_n_OSiMe_2_CH=CH_2_, vinyl 4.6mmol) with crosslinker hydrosilicone crosslinker HMS 301 (Me_3_Si(OSiMeH)_x_(OSiMe_2_)_y_OSiMe_3_, 0.928 g SiH 4.6mmol). A control elastomer **19** was prepared by adding diluted Karstedt’s catalyst solution (2 µL, concentration: 1 mg/ml) to 2.0 g of the batch mixture. No reinforcing agents were used. The mixture was then degassed by a vacuum desiccator, heated at 80 °C overnight. In a typical synthesis of MTDQ resin reinforced/filled silicone elastomer (10 wt% loading), the premixed base (1.8 g) and synthesized unreactive silicone resin (0.2 g) were added in a 12-well plate. Diluted Karstedt’s catalyst solution (2 µL, 20 ppm, concentration: 1 mg ml^−1^) was added to the mixture, followed by rapid mixing and degassing in a vacuum desiccator. The gelation was achieved in ~10 min after mixing at room temperature. The plate was then placed in a 80 °C oven overnight to achieve complete cure. Young’s modulus data and adhesion data to be found in Tables S3 and S4.

## 5. Conclusions

MTDQ resins of precise structure were readily synthesized by a combination the PR and Pt-catalyzed hydrosilylation reactions. A library of compounds comprised of linear, lightly branched and highly branched structures in 2-, 30- and 4-fold symmetry and ranging in MW from ~2000–~24,000 g mol^−1^ was prepared. Although increased branching led to higher viscosity of the oils due to intrachain interactions, when placed into a gel, such oils led to lower work of adhesion then lightly branched analogues of the resins. When selecting silicone oil structures to control rheological properties, increased molecules weight and branching provide excellent design criteria, but must be tempered such that M-rich oils to do phase separate from the D-rich elastomer network.

## Figures and Tables

**Table 1:** Work of adhesion of oil filled elastomers.

Series	Compound	MW (g mol^−^^1^)	Work of Adhesion (J/mm^2^)		
**Blank control elastomer**	**19**		0.3469		
			10% Loading	25% Loading	50% Loading
Small Dendron	**3**	2168	0.3833	0.5784	0.1578
	**4**	2836	0.3308	0.2976	0.1771
Linear	**14**	~6000	0.3394	0.5515	0.0254
	**15**	~9000	0.7993	0.5747	0.1350
	**16**	~12000	0.8969	0.5308	0.3985
**T(DM)_3_**	**8**	4523	0.8358	0.6011	0.2527
	**10**	6914	0.9700	0.7543	0.3372
	**12**	9107	0.9144	1.1406	0.3892
**T(TM_2_)_3_**	**9**	5858	0.5267	0.3680	0.0826
	**11**	8916	0.7703	0.6247	0.2320
	**13**	11776	0.8045	0.6415	0.6029
**T(TM_2_)_3_** Different core length	**17**	~6900	0.6712	0.4689	0.1616
	**18**	~14000	0.6928	0.6275	0.1297

## Supplementary Materials

The following are available online, Tables of viscosities of polymer oils **8**–**18** and Young’s moduli of gels containing the oils.
