# Supplementary material for: Hyperbranched Silicone MDTQ Tack Promoters
Source: Molecules. 2019 Nov 15;24(22):4133. doi: 10.3390/molecules24224133 (PMC6891585; doi:10.3390/molecules24224133)
Supplement: Supplementary file 1 [file molecules-24-04133-s001.pdf]

molecules

Hyperbranched Silicone MDTQ Tack Promoters

Sijia Zheng [1], Shuai Liang [1], Yang Chen [1] and Michael A. Brook [1]*

Supporting Information

Table S1 Viscosity of branched silicone oils.

Series	Compound	Symmetry	M_n (g mol^{-1})	Viscosity (Pa s)[a]
Linear	**14**	2-fold	~6000	0.050 [a]
	**15**	3-fold	~9000	0.076 [a]
	**16**	4-fold	~12000	0.098 [a]
**$T(DM)_3$**	**8**	2-fold	4520	0.059 [a]
	**10**	3-fold	6915	0.085 [a]
	**12**	4-fold	9105	0.102±0.0002
**$T(TM_2)_3$**	**9**	2-fold	5860	0.232±0.005
	**11**	3-fold	8915	0.241±0.005
	**13**	4-fold	11775	0.322±0.005
**$T(TM_2)_3$** Different core length	**17**	2-fold	6900	0.206±0.005
	**18**	2-fold	14000	0.336±0.005

[a]：The value is the same between three measurements

x

Table S2: Tack strength of oil filled elastomers

**Series**	**Compound**	**MW (g mol^{-1})**	**Tack Strength kPa)**		
Control elastomer	**19**		8.5204 +/- 0.0691		
			10% Loading	25% Loading	50% Loading
Small Dendron	**3**	2168.62	8.09623 ± 0.75656	8.09623 ± 0.75656	2.71439 ± 0.10792
	**4**	2836.01	7.46927 ± 0.15361	5.844826 ± 0.18693	2.77014 ± 0.22207
Linear	**14**	~6000	7.22925 ± 0.10181	5.40423 ± 0.0756	0.971 ± 0.09968
	**15**	~9000	11.20013 ± 0.7041	7.27092 ± 0.28806	2.26878 ± 0.23277
	**16**	~12000	12.26056 ± 0.79266	8.19801 ± 0.22796	4.47922 ± 0.06505
**T(DM)$_3$**	**8**	4523	9.6581 ± 0.10017	6.60664 ± 0.15648	3.09492 ± 0.08518
	**10**	6914	10.51651 ± 0.70786	8.17695 ± 0.36596	3.68183 ± 0.06609
	**12**	9107	11.68579 ± 0.20547	10.94327 ± 0.21773	5.21646 ± 0.13809
**T(TM$_2$)$_3$**	**9**	5858	7.26647 ± 0.44517	5.15376 ± 0.22379	2.02738 ± 0.11001
	**11**	8916	9.82008 ± 0.40213	7.8979 ± 0.30627	3.32983 ± 0.04178
	**13**	11776	9.99004 ±0.34274	8.95518 ± 0.10689	7.22283 ± 0.48322
**T(TM$_2$)$_3$** Different core length	**17**	~6900	8.54824 ± 0.16395	5.6636 ± 0.11569	2.76614 ± 0.05318
	**18**	~14000	8.01910 ± 0.19798	6.15555 ± 0.11348	2.47639 ± 0.17896

Table S3 Young's Modulus of silicone gels containing branched oils

**Series**	**Compound**	**MW (g mol^{-1})**	**Young's Modulus (MPa))**		
Control elastomer	**19**		0.4271 +/- 0.0201		
			10% Loading	25% Loading	50% Loading
Small Dendron	**3**	2168.62	0.349048 ± 0.019012	0.231311 ± 0.015289	0.095297± 0.004173
	**4**	2836.01	0.344211 ± 0.018276	0.234265 ± 0.012806	0.088409± 0.002946
Linear	**14**	~6000	0.31427 ±0.00564	0.10809 ±0.00307	0.07562 ± 0.00261
	**15**	~9000	0.32031 ±0.02203	0.18774 ±0.01457	0.07783 ± 0.00182
	**16**	~12000	0.34209 ±0.0297	0.2584 ±0.0166	0.10276 ± 0.00672
**T(DM)$_3$**	**8**	4523	0.22778 ± 0.00631	0.14821 ±0.00251	0.07736 ± 0.01436
	**10**	6914	0.2327 ± 0.0138	0.18091 ±0.00382	0.08205 ± 0.00676
	**12**	9107	0.30481 ± 0.01453	0.2143 ±0.00938	0.14269 ± 0.0044
**T(TM$_2$)$_3$**	**9**	5860	0.20461 ± 0.00386	0.14732 ±0.00406	0.1016 ± 0.01106
	**11**	8916	0.2555 ± 0.00497	0.2038 ±0.01421	0.09754 ± 0.00513
	**13**	11776	0.25318 ± 0.01587	0.25513 ±0.01568	0.1766 ± 0.00583
**T(TM$_2$)$_3$** Different core length	**17**	~6900	0.222201 ± 0.013069	0.139623±0.008126	0.096611±0.00783
	**18**	~14000	0.189452 ± 0.002805	0.123252±0.0052635	0.096517±0.00450
